# Translating an In-Class Deliberation Module to a Two-Year College Introductory Chemistry Course

**DOI:** 10.1021/acs.jchemed.5c00425

**Published:** 2025-07-18

**Authors:** Reni Joseph, David R. Brown, Sara A. Mehltretter, Katherine R. Knobloch, Anthony J. Roberts, Pamela Conners, Laura M. Wysocki

**Affiliations:** † Department of Physical and Engineering Sciences, 7546 St. Louis Community College, 3221 McKelvey Rd., Bridgeton, Missouri 63044, United States; ‡ Department of Chemistry, 501162 Southwestern College, 900 Otay Lakes Rd., Chula Vista, California 91910, United States; § Department of Rhetoric, 2719 Wabash College, 301 W. Wabash Ave., Crawfordsville, Indiana 47933, United States; ∥ Department of Communication Studies, 3447 Colorado State University, Fort Collins, Colorado 80523, United States; ⊥ Department of Sociology, Colorado State University, Fort Collins, Colorado 80523, United States; # Department of Communication Studies, 7573 Gustavus Adolphus College, 800 W. College Ave., St. Peter, Minnesota 56082, United States; ∇ Department of Chemistry, Wabash College, 301 W. Wabash Ave., Crawfordsville, Indiana 47933, United States

**Keywords:** First-Year Undergraduate/General, Environmental Chemistry, Interdisciplinary/Multidisciplinary, Public Understanding/Outreach, Communication/Writing, Problem Solving/Decision Making, Applications of Chemistry, Student-Centered Learning

## Abstract

In general chemistry, instructors have valuable opportunities to connect foundational chemistry concepts to larger societal issues early in a student’s academic career. Deliberation, a facilitated conversation that considers different perspectives on complicated and multidisciplinary issues, is one method to achieve this and has previously demonstrated positive effects at four-year institutions. However, the content demands within general chemistry can make this goal difficult to attain and two-year colleges can have a distinctly different environment from four-year colleges. Here, we discuss the transferability of a deliberation module about environmental contaminants to a Foundations of Chemistry course at a two-year college, including training recommendations, implementation suggestions, and positive outcomes. Student participants in the 2 h activity reported a high-quality experience during the deliberation, including considering others’ perspectives, and they showed motivation to take action to improve water quality. Moreover, the instructor for the course noticed that the activity helped to build community within the class and develop connections between current societal issues and the material they were learning in the course, in this case ion solubility.

## Graphical abstract

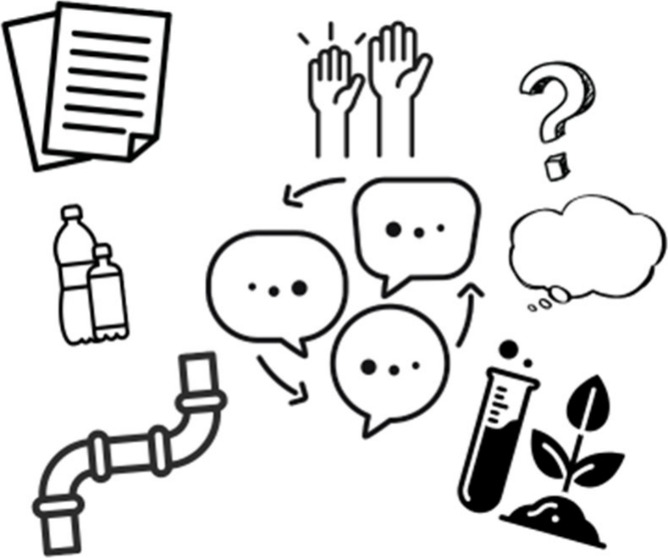

Early coursework in chemistry establishes foundational concepts and useful practices in the field, while also engaging students and building enthusiasm that can help them discover a sustained interest in pursuing further coursework. Many pedagogical efforts show that students appreciate connecting theoretical ideas with real-world applications^1–4^ and recent efforts in systems thinking highlight the role chemistry can play in complex problems in a societal context.^5–8^ We previously reported outcomes from a 2 h module using deliberation,^9^ which is a facilitated conversation that encourages participants to weigh different options in addressing the socio-scientific issue of water quality. Deliberation connects concepts learned in General Chemistry with the consideration of community stakeholders and different perspectives to come to a collective decision. Here, we describe how this module can be used in a two-year college environment.

Enrolling approximately 38% of the nation’s undergraduates,^10^ two-year colleges serve a myriad of purposes in the higher-education landscape. Their contributions range from preparing students to transfer to four-year institutions, to educating skilled technical workers for direct entry into the workforce, to graduating substantial proportions of health professionals, and more. Two-year colleges provide an economically advantageous option for many learners, as the average cost of tuition and fees for an in-district commuter at a two-year college ($3,990) is about 35% of what an in-state student pays for tuition and fees ($11,260) at a public four-year institution.^11^ Two-year colleges serve as entry points into higher education for many ethnic and racial minorities. In 2020, two-year colleges enrolled 51% of Hispanic undergraduates, 12% of Black undergraduates, and 42% of Asian undergraduates.^12^ Furthermore, two-year colleges offer 70% of dual enrollment courses,^13^ which are now available to more than 80% of public high school students. With open-access admissions policies, two-year colleges provide a pathway into the workforce for a wide swath of the American population pursuing growth and advancement through education.

Considering the host of academic programs requiring at least one chemistry course, continuing issues of retention in gateway STEM courses,^14–18^ along with the confluence of demographic and economic factors associated with two-year colleges, a curricular intervention that promotes student success, such as deliberation, plays an important role. However, the necessary content coverage in general chemistry, especially in courses used for transfer credit to other institutions, can make it difficult for instructors to find space in their syllabus to develop skills like science communication.

## Method

This deliberation was carried out at a public two-year college in the Midwest. The Foundations of Chemistry course has a lab component, is part of the transfer-guaranteed program, is suitable for students pursuing allied health careers, and serves as a prerequisite for General Chemistry. To the best of our knowledge, this is the first example of deliberation being implemented at this college, so students would be unfamiliar with the process. Data collected from this course was compared to a previously reported data set^9^ from General Chemistry courses at two residential liberal arts colleges (one all-male and one coeducational) in the Midwest, both of which support a cocurricular deliberation program. Further demographics related to participants can be found in the Supporting Information, with notable differences including a higher percentage of women, African American students, and students over the age of 24 in the two-year college data set when compared to the four-year colleges. This research was approved by the Institutional Review Board of Wabash College (IRB #1911181) and all participants provided informed consent for their survey responses to be included in the analyses presented here. No unexpected or unusually high safety hazards were encountered during this activity.

### Deliberation Module

The deliberation module focuses on environmental contaminants, specifically, water quality. At St. Louis Community College (STLCC), the instructor incorporated the activity alongside a discussion of solubility properties of ions. Students worked through practice problems using solubility rules and performed a lab with qualitative observations of the precipitation of ionic compounds in water. This placement in the syllabus formed a close connection between the concepts behind lead ion solubility and the real-world consequences of lead in the water in Flint, Michigan. To prepare for the deliberation, students read an article about issues the Flint community has faced as a consequence of changing their water source.^19^ They also read an article about a local issue of water quality: microplastics in the Missouri River.^20^ In pairing these articles, students have the opportunity to directly relate chemistry course content with a familiar, nationally recognized water quality emergency, while realizing that water quality is a pervasive issue for many communities, including their own.

The in-person deliberation module took place with small groups consisting of 4–6 students and a trained facilitator, who was not enrolled in the course. Because STLCC does not have a deliberation program on campus, two students who previously took the chemistry course and the instructor served as facilitators. Their training centered around an introduction to basic facilitation using resources^21,22^ that encouraged “passionate impartiality”^23^ to enable participant-driven conversation and included strategies to encourage all voices to be heard while focusing the activity on the issues at hand. These facilitators also received a detailed facilitator guide with suggested language to frame the issue, timing cues to keep the activity moving forward, and several questions to prompt discussion (see Supporting Information).

The deliberation began with discussing the importance of considering many perspectives and weighing several options. First, students engaged the perspectives of several stakeholders – both public and expert – in the complex issue of water quality. Next, they considered the benefits and potential drawbacks to three distinct options that could address water quality: enacting harsher penalties and regulations for those who do not protect the public; incentivizing positive behavior, including prevention, testing, and education programs; and investing in our society through infrastructure and training to address and prevent incidents. After discussing each option in turn, the group decided how our society should proceed with action steps. The final stage included a conversation about science and public policy, highlighting scientists’ role in engaging with the public.

### Assessment

Participants in the deliberation completed an online survey the week before the activity and another within a week after the activity. Over 2 semesters, 30 students were enrolled in the course and 24 students at the two-year institution completed both surveys. The surveys included Likert-scale questions that probed student experience with the deliberation, their civic and scientific attitudes, their knowledge about lead contamination in water, and their views on the role of the public and scientific experts in decision-making. In the postactivity survey, there were open-ended questions about their experiences during the deliberation and their views on environmental contaminants. A complete list of items in the surveys are in the Supporting Information. These surveys were also used to assess participants in General Chemistry using the same deliberation module at two residential four-year liberal arts colleges;^9^ the student responses across institutions were compared, with key data shown in Table 1.

**Table 1:** Means, Standard Deviations, and One-Way Analyses of Variance Across Institutions

	*M (SD)*			
Measure	Two-Year *N* = 24^c^	Four-Year (All Male) *N* = 90^c^	Four-Year (Mixed) *N* = 196^c^	*F* (2, 307)^c^
Evaluations^a^				
Analytic Quality	4.46 (0.56)	4.31 (0.61)	4.26 (0.54)	1.37
Democratic Quality	4.40 (0.58)	4.37 (0.49)	4.30 (0.47)	1.08
Motivation to Act	4.42 (0.72)^+^	3.86 (0.68)^+^	3.89 (0.69)^+^	16.27**
Opinion Change	3.29 (1.16)^+^	2.27 (0.73)^+^	2.39 (0.77)^+^	6.81*
Impacts^b^				
Knowledge	0.17 (1.74)	0.77 (1.93)	0.88 (2.00)	1.41
Understanding	0.38 (0.76)	0.28 (0.79)	0.33 (0.82)	0.03

a Evaluation measures are means of post-activity only questions about students’ experiences.

b Impacts are the average of the difference scores for students, calculated as the difference between their postsurvey response and their presurvey response.

c *p < 0.01; **p < 0.001;^+^represents a significant difference between the two-year college and the other institutions.

## Results

After their deliberations, students assessed the analytic quality of their discussion from “Very Dissatisfied” (1) to “Very Satisfied” (5) (3 items, α = 0.67). Students rated their experience highly, with a mean score of 4.46. They similarly rated the conversations’ democratic quality (5 items, α = 0.66) highly with a mean score of 4.40. Students were asked whether the conversation motivated them to act to improve water quality. Whereas 3 students said they felt the same as before, the large share (87.5%) said that they felt somewhat (*N* = 8) or much more motivated than before (*N* = 13). None reported feeling less motivated. When asked whether the conversation changed their view on environmental contaminants some reported their views were entirely (*N* = 1) or mostly the same as before (*N* = 6), but the majority (74.9%) said that their views changed somewhat (*N* = 6), a great deal (*N* = 7), or entirely (*N* = 4).

The pre- and postactivity surveys also asked about their knowledge of lead in the water using a summative scale of ten true or false items. A paired sample *t*-test showed no statistically significant change between the students’ preactivity and postactivity scores (*M*_post_ – M_pre_ = 0.17, *t*(23) = 0.48, *p* > 0.05). Students did, however, show a statistically significant increase (M_post_ – M_pre_ = 0.38, *t*(23) = −2.41, *p* < 0.05) in their sense that they understand chemistry in real-world contexts (3 items, α = 0.67) between the preassessment (M = 3.90, *sd* = 0.70) and postassessment (M = 4.28, *sd* = 0.79).

One-way analysis of variance (ANOVA) was used to test how changes at the two-year institution compared to its four-year counterparts. Results are provided in Table 1. No significant differences emerged in student assessments of analytic or democratic quality, though students at the two-year college saw significantly higher increases in their motivations to act and in their opinion changes compared to both four-year colleges. Looking at impacts, no differences emerged in knowledge or understanding.

Students were asked an open-ended question: “What did you learn from the deliberation(s) you took part in? Name up to 3 things.” The responses (*N* = 24) were qualitatively assessed using an inductive thematic analysis,^24^ with five themes emerging. More than half of the open responses conveyed learning about the issue discussed, with responses highlighting information about “microplastic[s] in rivers and landfills,” “learning new things about microplastics,” and “the water in Flint, MI.” The other four themes focused on considering different perspectives, taking action, civic connection, and scientists’ public identity. Demonstrating a learning outcome of deliberation,^25^ the taking perspective responses revealed that students encountered views and experiences different than their own: participants conveyed “there are many different options to addressing each issue”; “I learned important views and facts from my peers during this deliberation”; and that there were “difference[s] in opinions based on socioeconomic background.” A few responses encouraged the importance of taking action (e.g., “Ways I can be a help in my own community”), the recognition of civic education and knowledge for addressing public issues (e.g., “How important it is to know your representatives, local and more”); and the role for scientists in public problems (e.g., “I learned that Scientist[s] are very valuable to our society and world”).

Our previous assessment included institutions with cocurricular deliberation programs; STLCC relied on the instructor and former students with minimal training for implementation. Across all three institutions, we grouped first-time facilitators together, described as minimally trained, and we grouped facilitators who had experience in one or more previous deliberations as highly trained facilitators. Paired sample *t*-tests were run on subsamples of the data, split by facilitator experience, to determine if experience influenced outcomes. Both groups demonstrated significant gains in their chemistry knowledge and understanding of chemistry, with further details presented in the Supporting Information.

## Discussion

### Participant Survey Reflections

Deliberation and its collaborative approach to applying knowledge to address socio-scientific issues is unlike typical pedagogy in the classroom focused on theoretical problem-solving paired with exams. In addition, the minimally trained facilitators in this situation did not come to the table with great familiarity with the topic nor with the deliberation process. Nonetheless, our results demonstrate positive outcomes for participants, both in terms of quality and impacts. This is critical to the transferability of the activity because a deliberation program is not available at most institutions. Deliberation relies on trained, “passionately impartial” facilitators to foster more democratic discussion; training and appropriate materials for the facilitator and participants is key to success.^23^ Especially in a course at a two-year institution where students have such diverse lived experiences to contribute to discussions, high marks on analytic and democratic quality, alongside a recognition of different perspectives in open-ended responses, are encouraging.

Most importantly, we find a strong positive change in participants’ motivation to act after deliberating about environmental contaminants. Not only did survey responses indicate the importance of taking action, but the instructor (R. J.) also noticed enthusiasm among their students. Students were excited to discuss a shift toward reusable water bottles in the weeks following the deliberation and some reported having conversations with their friends about what they learned. These actions, combined with the overall reported increase in motivation to act, represent a link students made between the chemistry concepts in class and their own experiences. The Foundations of Chemistry course faces issues of poor retention and low student engagement, especially given that many of those enrolled are part-time students, with full-time jobs and families to support outside of the classroom. The applicability of the course content to real-world problems can serve as motivation, helping students stay connected with the subject, despite challenges.

Knowledge gains among participants at the two-year college did not mirror what we saw at four-year institutions; while participants reported learning about issues like microplastics and lead in the open-ended questions, there was not a statistically significant gain in correctness when responding to statements about lead in water. This may stem from a difference in execution of the deliberation module between the two- and four-year colleges. Participants at the four-year college were assigned a short perspective from a scientific expert on environmental contaminants,^9^ which was omitted at the two-year college at the instructor’s discretion. Though research on the four-year colleges indicated that the amount and type of reading did not have an independent effect on student learning,^9^ the two-year college findings suggest that the readings may impact learning outcomes. In addition to strengthening our results by increasing the sample size of participants at two-year institutions, future research should continue to explore how the presence of distinct informational materials impact knowledge gains. Despite this, the instructor felt that the deliberation helped students to understand the concept of ion solubility on a practical level when they could see the real-world implications of the theoretical solubility rules.

### Instructor Reflections on Challenges and Opportunities

To complement what we have learned from student surveys, the instructor (R. J.) adds insight into the challenges of implementation at two-year colleges and some opportunities. Two anticipated challenges of first-time implementation are making space in the already-crowded syllabus for general chemistry and unfamiliarity with the practice of deliberation. In this case, we paired the deliberation with existing course content, specifically a traditional reactions of ions lab that relates to solubility. Though this jettisoned some in-class problem-solving practice, students seemed to enjoy the deliberation and could see the impact of what they were learning, making this trade-off worthwhile. While many STEM instructors have experience with group work, the structure of deliberation is different, and unfamiliarity can be a barrier to adoption. After a brief introduction to the ideas behind deliberation,^26^ the instructor used the provided resources^21,22^ to become familiar with the facilitator role and responsibilities. Although peer facilitators are recommended, circumstances necessitated that the instructor serve as a facilitator in one group, so they also used the facilitation guide. With this support, they felt prepared as both an instructor and a facilitator.

Two unanticipated challenges arose during implementation. First, recruiting peer facilitators who were not current students in the course was difficult. Unlike four-year colleges that have upperclassmen who might serve in this role, two-year colleges have a smaller pool of students to draw from and the student-faculty relationship may not be maintained after a course is complete. In this case, two former students served as facilitators. A second issue that arose is lack of space. Deliberation necessitates listening carefully and having voices heard, and with multiple groups deliberating simultaneously, it is optimal to have more space than a typical lecture room provides. With a Saturday course time, other rooms were unavailable, so the hallways provided adequate space for discussion. The logistics of implementation are important for student experience and comfort during the deliberation.

After experiencing the deliberation firsthand, the instructor saw it as an opportunity to build and strengthen relationships that can be difficult to develop at a two-year institution. While residential liberal arts colleges and larger four-year institutions provide many opportunities for students to interact and form relationships outside the classroom, the vast majority of students at two-year colleges commute to campus and only interact with other students in the classroom environment. This can make it difficult to foster a collaborative classroom culture that empowers students to participate freely, make mistakes, and grow through the learning process. After the deliberation activity, the instructor saw noticeable improvement in students’ willingness to ask the instructor or a peer for help and students’ interactions with each other. For example, several students began the semester barely speaking to their lab partner during experiments, but after the deliberation, they worked more effectively as a team. The deliberation invited greater student engagement than other typical classroom activities and the students found common ground with their peers, despite their varied academic preparation and life experiences. Classroom culture and sense of belonging^14–18^ contribute to student success in gateway STEM courses, so these peer relationships are important to develop.

## Conclusion

While previous work has shown positive outcomes from the use of a deliberation module in general chemistry at four-year colleges,^9^ here we have demonstrated the transferability of deliberation to an analogous introductory course at a two-year institution, which has a student population with very different demographics and experiences. Even without a cocurricular deliberation program on-site, the resources allow for adequate facilitator training to deliver participants an experience with high analytic and democratic quality. After deliberating on how society should address environmental contaminants, participants reported feeling more motivated to act. Instructor reflections reveal student enthusiasm around water quality and an appreciation for the connection of class concepts to a real-world issue. Challenges for implementation were overcome and deliberation added value to the course.

The differences in results between two-year and four-year institutions suggest that deliberation may play an important role for establishing student relationships at two-year institutions. Because of this critical community-building potential, future directions include moving the deliberation earlier in the semester to foster a positive class culture. Given its potential impact on students’ sense of belonging and understanding of the real-world applications of chemistry, we also hope to investigate the effect an early deliberation module might have on student retention in this introductory chemistry course.

## Supplementary Material



## Notes

Participant Demographic Table; Facilitation Guide; Survey Measures; Facilitator Training Comparison (PDF)
