# Supplementary material for: Translating an In-Class Deliberation Module to a Two-Year College Introductory Chemistry Course
Source: J Chem Educ. 2025 Jul 18;102(8):3691–5. doi: 10.1021/acs.jchemed.5c00425 (PMC12355903; doi:10.1021/acs.jchemed.5c00425)
Supplement: Supplementary file 1 [file ed5c00425_si_001.pdf]

Supporting Information

Translating an In-Class Deliberation Module to a Two-Year College Introductory Chemistry Course

Reni Joseph[1], David R. Brown[2], Sara A. Mehltretter[3], Katherine R. Knobloch[4], Anthony J. Roberts[5], Pamela Conners[6], Laura M. Wysocki[7]*

[1]Department of Physical and Engineering Sciences, St. Louis Community College, Bridgeton, MO 63044, USA

[2]Department of Chemistry, Southwestern College, Chula Vista, CA 91910, USA

[3]Department of Rhetoric, Wabash College, Crawfordsville, IN 47933, USA

[4]Department of Communication Studies, Colorado State University, Fort Collins, CO 80523, USA

[5]Department of Sociology, Colorado State University, Fort Collins, CO 80523, USA

[6]Department of Communication Studies, Gustavus Adolphus College, Saint Peter, MN 56082, USA

[7]Department of Chemistry, Wabash College, Crawfordsville, IN 47933, USA

*To whom correspondence should be addressed: wysockil@wabash.edu

Table of Contents

Participant Demographic Table S2

Facilitation Guide S3

Survey Measures S10

Facilitator Training Comparison S15

Demographic Data, Two-Year College, n=24

Table S1. Demographic Data, Deliberation Participants, Two-Year College

Gender		Race	
Man	2	African American	10
Woman	22	White, Non-Hispanic	10
Other	0	All Other Races	3
**Age**			
Under 18	0		
18-24	5		
25-34	10		
35-44	8		
45 or older	1		
**Party Affiliation**		**Ideology**	
Democrat	7	Liberal	3
Republican	1	Moderate	13
Independent/No Party	4	Conservative	7
Don't Know/Decline	12		

Demographic Data, Four-Year Colleges, n=279

Table S2. Demographic Data, Deliberation Participants, Four-Year Colleges

Gender		Race	
Man	155	African American	6
Woman	113	Asian American	11
Other	7	Hispanic or Latinx	9
		White, Non-Hispanic	222
		Other	5
		More than one race	17
**Age**		**Science Major**	
18	106	Science Major	253
19	111	Non-Science Major	21
20	43	**Chemistry Major**	
21	8	Chemistry Major	67
22	6	Non-Chemistry Major	207
24	1		
69	1		
**Party Affiliation**		**Ideology**	
Democrat	101	Liberal	104
Republican	80	Moderate	80
Independent/No Party	25	Conservative	91
Don't Know/Decline	73		

This guide was created with contributions from Sara A. Mehltretter Drury (Wabash College), Laura M. Wysocki (Wabash College), Arthur Equihua (Wabash College '20), Hayley Blonsley (Wabash College), Amanda Nienow (Gustavus Adolphus College), and Pamela Conners (Gustavus Adolphus College).

Environmental Contaminants Deliberation Facilitation Guide

Time: 2-2.25 hours

Activity Description:

You will participate in a deliberation about environmental contamination, with a focus on water quality. Contaminants in our soil and water can have significant, dangerous impacts on our environment and our overall health. Recently in the United States, it seems like issues of contaminants in the soil and water most impact those who are already struggling to make ends meet. In this lab, you will deliberate about how to counter effects and prevent future incidents.

A deliberation is a rational process through which participants consider different perspectives in order to collaboratively arrive at a decision. Your conversation will be guided by a trained facilitator, who will ask you to examine the problem of water and soil contaminants carefully and weigh the benefits and tradeoffs of different approaches for addressing it.

Preparation Work for students and facilitators

All students:

- Watch video: https://www.cbsnews.com/news/the-flint-water-crisis-a-loss-of-trust/
- Read news local news stories: https://www.columbiamissourian.com/news/local/how-microplastics-are-threatening-water-and-land-across-missouri/article_bd4092fc-ec0b-11ec-a059-2b00303c349b.html

Ideally, deliberation groups should be a minimum of 5 students and a maximum of 10.
One facilitator is needed per group.

Facilitation Plan

Agenda (2 hours):

1. Opening Framing (5 minutes)
2. Introductions in Groups (5 minutes)
3. Stage 1: Gaining Perspective of the Different Roles in Society (20 minutes)
4. Stage 2: Deliberation with group (45 minutes)
5. *Break (5 minutes)*
6. Stage 2 continued: Deliberation with group (20 minutes)
7. Stage 3: Science and Civics (20 minutes)
8. Conclusion (5 minutes)

1. Opening Framing of the Deliberation.
***As class begins*: Instructors should introduce the deliberation activity; sample remarks are presented here.** (5 min)

Instructor:
This week, our class will be investigating the issue of environmental contaminants from a deliberative perspective. Public deliberation is a group communication and critical thinking process that enables us to consider public issues from many perspectives, weigh options, and then decide what should be done. Deliberations bring together facts and experiences from many people, including scientists, government officials, members of the public, and more. Deliberation encourages the *slow* consideration of a problem so that multiple perspectives can be explored through different actions, each with benefits and tradeoffs.

Today, you will be taking part in a deliberation. Your group will engage a variety of public experiences of the issue of contaminants in the water and soil, consider benefits and tradeoffs of actions, and ultimately, decide what should be done.

We'll turn it over to your small group facilitators so they can begin this exercise.

[*Instructor and may leave the room or sit off to the side. Instructor should not participate in or interrupt the deliberation. Students may feel less likely to deliberate openly if the instructor is "listening" or contributing. The goal is that students are able to discuss in a peer-to-peer setting.]*

2. Introductions in Groups (10 min)

[Facilitators should introduce themselves]
Hello, my name is ____________, and I will be the facilitator today. My role is to guide the conversation in an impartial, neutral way.

There are a few guidelines that we would like to lay out that will make this deliberation successful.

- First, we encourage everyone to participate, and no one or two individuals should dominate.
- Second, listen to others. Listening is as important as speaking in a deliberation.
- Third, keep an open mind to new ideas. Avoid conclusions until we have discussed all the options.
- Fourth, disagree with curiosity; the purpose of deliberations is to learn about the issue and others' perspectives.
- Finally, please silence your cell phones.

[note to facilitators: please practice this ahead of time so that you can deliver/read it with energy and enthusiasm]

Today we will take time to consider the public problem around environmental contaminants in our water and soil. The issue of contaminants is a complex scientific issue that involves various

types of scientific expertise and knowledge. It also is a complicated **public** issue, which involves many different stakeholders.

In fact, this issue is so complicated, we might call it a **wicked problem**. Wicked problems are public issues with many stakeholders that are hard to understand and challenging to address through single actions. Furthermore, any action step or solution to a wicked problem has tradeoffs that have to be managed—no action is perfect, and so part of the work of **deliberation** is to weigh the **benefits** and **drawbacks** of different actions.

So today, we'll explore a variety of potential approaches, and spend time working through different aspects of the issue. Ultimately, we want you to use this experience to decide how best to manage the problem of contaminants in the environment.

In the first stage of the deliberation, you'll think about different perspectives on this problem, using the readings you did before today's lab period to inform our understanding of the issue.

In the second stage of the deliberation, you will then consider <u>three</u> potential approaches to addressing and managing this problem. After weighing different options you will then decide what should be done.

In the final stage, you'll consider the role of scientists in addressing scientific public issues and in engaging the public.

Before we begin, let's introduce ourselves by sharing our names.

<u>3. Stage 1: Gaining Perspective of the Different Roles in Society (20 minutes)</u>

Our first task today is to think about this issue as a public, wicked problem. You all watched a video on Flint, Michigan, and read the news article about contamination in ___________________ ___________________[*insert city/state from readings*]. These news sources talk about the issue from the public perspective. We'll focus our conversation for the next 20 minutes on the different stakeholders around this issue.

Let's talk more about what we learned about the issue.

Questions:

- Let's focus first on <u>public perspectives of the issue,</u> or non-scientist perspectives. Based on the news reports who is concerned about this issue? Who does this issue impact?
    - *Have them get specific—should produce different professions and community groups. Should include community-perspectives as well as experts.*
    - For each stakeholder mentioned, what information do they bring to the issue? What should we be aware of?
    - For each stakeholder mentioned, what are their concerns about the issue?
    - Are there stakeholders in the public missing from these news reports?

- Now let's focus on <u>the expert perspectives you read.</u>

- First, let's go around and share a brief summary of what you read. What does your perspective bring to the issue?
- Some of you read the same perspectives, so if you have someone who read what you read, please speak up immediately after them.
- *[Once everyone has shared]* What stakes do these experts have in the problem of contaminants?

- As you think about the different stakes amongst members of the public, government, and scientific experts, are there any conflicts or tensions amongst the concerns we've discussed?
- Are there questions that remain about the facts or circumstances of this issue? By this we mean what the problem is, not what should be done, because we'll be deliberating what should be done
    - *[Facilitator: you might list the questions and then ask your if anyone in your group can answer from their knowledge or perspective reading, or if the information is not known for the members of the group.]*

4. Stage 2: Deliberation with group (60 minutes)
Facilitator: Now that we've thought more about the information and perspectives on the issue, let's turn to deliberating about different ways of approaching and addressing the problem.

Many communities are grappling with the issue of contaminants in the environment, including Flint, Michigan; [*insert city/state from readings*]. Today, we're going to deliberate how we can best protect against future issues with contaminants in our environment. To help our discussion, we will address this problem by considering three options, and then we will decide which are the best actions for the future.

Does everyone have a copy of the guide that they can look at? *[Facilitators can give extra guides to those who may not have a paper version of the "Placemat" guide].*

As you see on this chart, each option suggests a different focal approach to the problems of contaminants. The **options are not entirely exclusive; for example, multiple actions from one or two approaches could be selected.** However, we know that in addressing public problems, there are limited resources of time and personnel. So we have to make choices **about what is most important.** Today, we will discuss these options to make a better decision about which are the best possible actions going forward.

We'll spend about 15 minutes on each option, to highlight possible actions, benefits, and tradeoffs. Then, we'll spend time talking about what should be done in the future.

Option One: Enact harsher penalties and regulations for those who do not protect the public (15 min)

Facilitator: Could one of you please read the first option?

Participant Reads: *This option suggests that companies and governments do not always respond ethically to issues of contaminants in the soil. Harsher regulations and penalties may decrease future incidents.*

Facilitator: Thank you.

- What are some initial thoughts to this option?
- Who would benefit from this option?
- Are there any actions you think would be a good response?
- In addition the actions listed, what else might be done under this approach?
- What are potential drawbacks or concerns about this option?
    - One possible drawback is that there are already laws and regulations in place, but incidents continue to happen. Do you believe harsher penalties make an impact?
    - Will penalties foster greater accountability in companies?
- Can any of the drawbacks be addressed or managed to increase the benefit?
- What seems to be most important reason for pursuing this approach? *[Or, why might someone be in favor of pursuing this approach]*

*[At the end, the facilitator should give a **brief** (20-30 second) summary of what the group has discussed, reviewing benefits, tradeoffs, and any tensions you've heard, before moving to the next approach]*

Option Two: Incentivize positive behavior (15 min)

Facilitator: Could one of you please read the second option?

Participant Reads: *This option assumes that contaminants will continue to cause problems for U.S. communities, and places a funding priority on prevention, testing, and education programs.*

Facilitator: Thank you.

- What are some initial thoughts to this option?
- Who would benefit from this option?
- Are there any actions you think would be a good response?
- In addition the actions listed, what else might be done under this approach?
- What are potential drawbacks or concerns about this option?
    - One possible drawback is that this doesn't help places like Flint and East Chicago (add in communities close to you) who have been impacted. How can we balance protecting future risk with helping those already suffering?
- What seems to be most important reason for pursuing this approach? *[Or, why might someone be in favor of pursuing this approach]*

*[At the end, the facilitator should give a **brief** (20-30 second) summary of what the group has discussed, reviewing benefits, tradeoffs, and any tensions you've heard, before moving to the next approach]*

Option Three: **Invest in our society to address and prevent incidents (15 min)**
Facilitator: Could one of you please read the third option?
Participant Reads: *This option assumes contaminants can be reduced or prevented, and places a priority on developing better infrastructure and training others to respond quickly and ethically to incidents.*

Facilitator: Thank you.

- What do you think about this option?
- Who would benefit from this option?
- Are there any actions you think would be a good response?
- In addition the actions listed, what else might be done under this approach?
- What are potential drawbacks or concerns about this option?
    - One drawback is that contaminant issues can be hard to predict. How do we balance that uncertainty with the cost of investing in systems?
- What seems to be most important reason for pursuing this approach? *[Or, why might someone be in favor of pursuing this approach]*

*[give a **brief** (20-30 second) summary of what the group has discussed, reviewing benefits, tradeoffs, and any tensions you've heard, before moving to the next approach]*

Facilitator: Thank you. We're going to take a 5 minute break. Feel free to stretch or use the restroom. Please be ready to discuss again at ____[time].

5. Break (5 min)

6. Prioritizing Actions / Decision-Making (20 min)
Facilitator: Now that we have discussed the three options, let's bring everything together and weigh our decisions.

[facilitator should give a summary of the three approaches—be careful not to offer conclusions, but rather key ideas of what the group has said what should be done]

- Now that we've talked about three different options, are there other possibilities exist for actions?
- Thinking across all the options, what actions or priorities seem most important to do?
    - Why are these so important to addressing this issue?
    - What are the tradeoffs of these actions or priorities? Can the tradeoff be managed, or minimized? Or, are we willing to live with the tradeoff?
- Who needs to be involved in addressing this problem?
    - [[if they point only to institutions/large entities]] Are there ways that individuals can take part in addressing this issue?

- How can the public be involved?
    - How can science professionals take part in addressing this issue?
- How should our society proceed in the future to address this problem?

Facilitator: *[provide a brief summary of the conversation]*

7. Stage 3: Science and Civics (20 minutes)

Facilitator: Now that we've talked about this problem, let's take a step back and talk about science and public policy.

- What makes scientific wicked problems so hard to discuss?
- Do you feel like scientists and experts do a good job communicating with the public? Why or why not?
- *<<If they talked about education or the need for better information in their answers>>* You mentioned scientific communication and education. How can scientists communicate these ideas more effectively?
    - What strategies would you use, as a science student, to discuss these ideas in your communities if they faced environmental contaminants?
- *<<If they DID NOT talk about education or the need for better information>>* How might scientists contribute to addressing this problem?
    - How might scientists encourage more effective communication?
    - What strategies would you use, as a science student, to discuss these ideas in your communities if they faced environmental contaminants?

- What are positive ways that scientists engage with the public?
- What are positive ways that the public engages with science and scientists?
    - What are different public engagement strategies you have observed?
    - What has frustrated you about public engagement on science issues?

- How can the public be more involved in decision making for public issues relating to science?
- How can scientists be more involved in decision making for public issues relating to science?
    - In your view, who needs to be involved in these sorts of public issues?

- Based on our conversations in this deliberation, what will you take with you as you think about scientists and public engagement?

[Facilitator should offer a summary at the end, and thank everyone for attending]

Survey Measures

Analytic Quality (from Knobloch, Gastil, Reedy, & Walsh, 2013)
Items:

1. Do you believe that you learned enough in the deliberation activities to have an informed opinion about the issues discussed?
2. Did this deliberation(s) encourage you to weigh the most important arguments and issues relating to the deliberation topic?
3. Did the conversation help you understand different people’s perspectives on this issue?

Response Options:

1. Definitely Not
2. Probably Not
3. Unsure
4. Probably Yes
5. Definitely Yes

Democratic Quality (from Knobloch, Gastil, Reedy, & Walsh, 2013)
Items:

1. How often during the deliberation did you feel you had sufficient opportunity to express your views?
2. When others expressed views different from your own during the deliberations, how often did you consider carefully what they had to say?
3. How often during the deliberation do you feel that other participants treated you with respect?
4. How often during the deliberation did you have trouble understanding or following the discussion? (reverse coded)
5. How often during the deliberation did you feel pressured to agree with something that you weren’t sure about? (reverse coded)

Response Options:

1. Never
2. Rarely
3. Occasionally
4. Often
5. Almost Always

Knowledge Items
Items:

1. Oxidation of lead pipes increases the metal in the solution.
2. All lead compounds dissolve in water.

3. Boiling water reduces metal contaminants.
4. Sitting water generally has more contaminants than flowing water.
5. Children have a relatively higher risk of health issues associated with lead than adults.
6. Lead is still used widely in commercial products.
7. Lead accumulates in the body over time.
8. Increased lead exposure can lead to neurological issues.
9. Lead poisoning always results from a recent high exposure of lead.
10. Dangerous exposure to lead is primarily through the skin.

Response Options:

1. I know it is correct
2. I think it is correct
3. I am unsure if it is correct or incorrect
4. I think it is incorrect
5. I know it is incorrect

Interest in and Enthusiasm for Chemistry

Item: In this section, we'd like to ask you some questions about your knowledge of chemistry. Please reply to the following questions with how much you presently understand the topic.

1. Enthusiastic about chemistry
2. Interested in discussing chemistry with friends or family
3. Interested in taking additional classes in this subject
4. Planning to take additional classes in this subject

Response Options

1. Not at all
2. A little
3. Somewhat
4. A lot
5. A great deal

Confidence in Chemistry Abilities

Item: In this section, we'd like to ask you some questions about your knowledge of chemistry. Please reply to the following questions with how much you presently understand the topic.

1. Confident that I understand chemistry
2. Confident that I can do chemistry

3. Comfortable working with complex ideas in chemistry

Response Options
1. Not at all
2. A little
3. Somewhat
4. A lot
5. A great deal

Understanding of Chemistry
Item: In this section, we'd like to ask you some questions about your knowledge of chemistry. Please reply to the following questions with how much you presently understand the topic.
1. Chemical principles relating to water and environmental quality
2. How ideas we explore in chemistry relate to ideas I have encountered in other science classes
3. How studying chemistry helps people address real world issues

Response Options
1. Not at all
2. A little
3. Somewhat
4. A lot
5. A great deal

Internal Scientific Efficacy (adapted from Morrell, 2003)
Item:
1. I feel confident in my ability to make decisions about community issues that deal with science.
2. I think that I am more informed about the ways that science influences political decisions than most people.
3. I consider myself well qualified to take part in public decision making related to scientific issues.
4. People like me don't have any say in government decisions that relate to science.
5. Public officials listen to people like me when making decisions about scientific issues.
6. There are many ways for members of the public to influence government decisions related to science.

Response Options
1. Strongly Disagree
2. Disagree
3. Neutral
4. Agree
5. Strongly Agree

External Scientific Efficacy (adapted from Morrell, 2003)
Item:
1. People like me don't have any say in government decisions that relate to science.
2. Public officials listen to people like me when making decisions about scientific issues.
3. There are many ways for members of the public to influence government decisions related to science.

Response Options
1. Strongly Disagree
2. Disagree
3. Neutral
4. Agree
5. Strongly Agree

Opinion Change
Item: How did the class deliberation shape your opinion on the issue of environmental contaminants?

Response Options
1. My views are entirely the same as before
2. My views are mostly the same as before
3. My views changed somewhat
4. My views changed a great deal
5. My views changed entirely

Motivation to Act
Item: After our discussion, how motivated are you to take action related to environmental contaminants?

Response Options
1. I am much less motivated to act than before

2. I feel somewhat less motivated to act than before
3. I feel the same as before
4. I feel somewhat more motivated to act than before
5. I feel much more motivated to act than before

Reading Assignment Completion
Item: How much of the assigned readings did you complete prior to class? This question, like all others on this survey, will be used solely for research purposes, and your instructor will not have access to your answer.

Response Options
1. I read everything carefully.
2. I read most of it.
3. I skimmed the readings.
4. I did not do the assigned readings.

Facilitator Training Comparison

Paired sample t-tests were run on subsamples of the data, split by facilitator experience, to determine if experience influenced outcomes. Results are presented in Table S3. Both groups demonstrated significant gains in their chemistry knowledge, understanding of chemistry, and internal scientific efficacy, though only students with highly trained facilitators saw significant increases to their external scientific efficacy. Independent sample t-tests were further used to determine differences in outcomes based on facilitator experiences. Results are presented in Table S4. Students who participated in discussions facilitated by minimally trained facilitators reported higher analytic quality and greater changes to their motivation to act, opinion about environmental contaminants, and their understanding of chemistry principles than participants with highly trained facilitators. Students with highly trained facilitators saw significantly higher changes to their external efficacy and approached significantly higher changes to their knowledge. These results indicate that minimally trained facilitators can produce positive outcomes for students, though facilitator experiences does appear to impact the level and types of change. Future research should continue to explore how variations in facilitation style and facilitation experience impact outcomes, but our results indicate that classrooms can benefit from deliberative discussions without the presence of highly trained facilitators.

Table S3. Pre- and Post-Activity Comparison of Means Across Facilitator Experience at Two- and Four-Year Colleges

Measure	Minimally Trained (N = 158)			Highly Trained (N = 137)		
	Pre M (SD)	Post M (SD)	t	Pre M (SD)	Post M (SD)	t
Knowledge	6.234 (1.753)	6.899 (2.026)	-4.327***	6.029 (1.948)	7.037 (1.968)	-6.001***
Understanding	3.282 (.730)	3.708 (.703)	-6.873***	3.320 (.686)	3.552 (.740)	-3.296***
Internal Efficacy	3.196 (.651)	3.555 (.649)	-6.912***	3.110 (.780)	3.482 (.708)	-6.362***
External Efficacy	3.114 (.645)	3.141 (.687)	-.528	2.881 (.684)	3.127 (.690)	-4.198***

Note. t-score based on paired sample t-test. $^{***}p < .001$

Table S4. Means and Difference Score Comparisons by Facilitator Experience

Measure	Minimally Trained	Highly Trained	
	M (SD)	*M (SD)*	*t*
Evaluations (N = 301)			
Analytic Quality	4.354 (.550)	4.217 (.569)	2.112**
Democratic Quality	4.344 (.476)	4.293 (.476)	0.905
Motivation to Act	3.98 (.771)	3.83 (.638)	1.888**
Opinion Change	2.55 (.895)	2.30 (.719)	2.709**
Impacts (N = 294)			
Knowledge	0.665 (1.930)	1.007 (1.965)	-1.508^{+}
Understanding	.426 (.779)	.232 (.825)	2.073**
Internal Efficacy	.359 (.652)	.372 (.685)	-.175
External Efficacy	.027 (.653)	.246 (.685)	-2.800**

Note. t-score based on independent sample t-test. $^{+}p < .10$, $^{**}p < .01$
